# Analyses of 2-DEG characteristics in GaN HEMT with AlN/GaN super-lattice as barrier layer grown by MOCVD

**DOI:** 10.1186/1556-276X-7-141

**Published:** 2012-02-20

**Authors:** Peiqiang Xu, Yang Jiang, Yao Chen, Ziguang Ma, Xiaoli Wang, Zhen Deng, Yan Li, Haiqiang Jia, Wenxin Wang, Hong Chen

**Affiliations:** 1 Beijing National Laboratory of Condensed Matter Physics, Institute of Physics, Chinese Academy of Sciences, No. 8, 3rd South Street, Zhongguancun, Haidian District, Beijing, 100190, People's Republic of China

## Abstract

GaN-based high-electron mobility transistors (HEMTs) with AlN/GaN super-lattices (SLs) (4 to 10 periods) as barriers were prepared on (0001) sapphire substrates. An innovative method of calculating the concentration of two-dimensional electron gas (2-DEG) was brought up when AlN/GaN SLs were used as barriers. With this method, the energy band structure of AlN/GaN SLs was analyzed, and it was found that the concentration of 2-DEG is related to the thickness of AlN barrier and the thickness of the period; however, it is independent of the total thickness of the AlN/GaN SLs. In addition, we consider that the sheet carrier concentration in every SL period is equivalent and the 2-DEG concentration measured by Hall effect is the average value in one SL period. The calculation result fitted well with the experimental data. So, we proposed that our method can be conveniently applied to calculate the 2-DEG concentration of HEMT with the AlN/GaN SL barrier.

## Introduction

The GaN-based high-electron mobility transistor (HEMT) is a promising research subject because of the expected advantages for the realization of electronic devices for high-power and high-temperature operations [1-5]. There are also applications as bio-sensors for detection of bacteria, DNA, and so on. The Al_0.3_Ga_0.7_N/AlN/GaN structures achieved a two-dimensional electron gas (2-DEG) mobility of 2,185 cm^2^/V·s at room temperature with the carrier density of 1.1 × 10^13 ^cm^-2 ^which was deposited by metal-organic chemical vapor deposition (MOCVD) on sapphire substrate [6]. However, the relatively low carrier sheet density with AlGaN/GaN structures hinders the further development of device process. Although the sheet carrier density can be further improved by increasing the Al content in the ternary barrier layer, this increases the strength of polarization; on the other hand, increasing the Al content in the ternary layer may deteriorate the quality of AlGaN due to large lattice mis-match, and ternary alloy scattering in the hetero-structure resulted in poor transport properties [7]. In contrast, the growth of AlN barrier layer on GaN channel enhances the transport properties. Introducing nano-structures into the HEMT epi-layers can enhance the device performance. For example, recent reports have shown that by replacing the conventional AlGaN barrier layers with AlN/GaN super-lattices composed of several-nanometer-thick binary alloys, electric field induced by macroscopic polarization is much stronger, and the sheet carrier density is higher [8]. The AlN/GaN SLs have stronger polarization effect and larger conduction band offset than the hetero-structure of AlGaN/GaN, and the SLs have deeper triangular quantum well.

The carriers in the hetero-interface are confined two-dimensionally and have stronger quantum effect than hetero-structure of AlGaN/GaN as sheet carrier density, which makes more density of 2-DEG and higher mobility in GaN-based HEMT device possible. Despite excellent transport properties, the described AlN/GaN structures have an obvious drawback: the 2-DEG electron channel lies very close to the surface which makes it very sensitive to any process occurring at the surface of the sample. Compared with AlN as barrier, the 2-DEG electron of HEMT with the AlN/GaN SL barrier has strong anti-jamming because it has several similar 2-DEG electron channels which reduce any influence from surface by several times, and the sheet carrier density of HEMT with the SL barrier is much higher than AlGaN as barrier.

The purpose of this paper is to present detailed calculations about HEMT with the SLs as the barrier layers. There are many studies on growing the HEMT with SL barrier but there are few reports on calculations about the sheet carrier density of hetero-structure with SLs as barrier. As the energy band of HEMT with SL barrier is analyzed, we assume that the 2-DEG will be formed in every SL period if the thickness of AlN is beyond the critical thickness (*d*_0_) which is the least limit thickness of AlN barrier to form the 2-DEG. We also consider that the 2-DEG density in each SL period is equal since the SLs have periodicity and tunnel impenetration. We deduce the equation which can be applied to calculate the 2-DEG density in GaN/AlN/GaN hetero-structures and calculated the 2-DEG density with the data of our SLs, and at the same time, the calculated results were compared with experimental data.

## Experimental details

**Figure 1:**
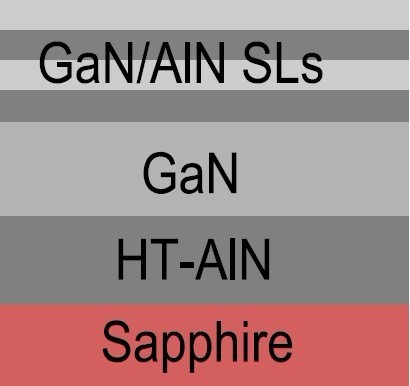
**Schematic diagram of GaN/AlN HEMT**. Area shaded in light and dark grey stripes, GaN/AlN Sls; light grey, GaN; dark grey, HT-AlN; red, sapphire.

AlGaN/GaN hetero-structures were grown by MOCVD on (0001) sapphire substrates using AlN/GaN SL (4 to 10 periods) structures as barrier layers where the top-most layer was GaN. Trimethylgallium, trimethylaluminum, and ammonia were used as Ga, Al and, N precursors, respectively. The schematic diagram is shown in Figure 1. Growth conditions of semi-insulated GaN buffer of all samples were the same and they had no intended doping. The equivalent Al composition of the quasi-alloy can be changed by adjusting the thicknesses of AlN and GaN layers. High-resolution X-ray diffraction was performed to measure the thickness of AlN/GaN SLs of the samples using Bede D1 system. The equivalent Al composition is defined as Equation 1:

(1) $$Al\%=\frac{d_{\text{AlN}}}{d_{\text{AlN}}+d_{\text{GaN}}}\times 100\%$$

**Figure 2:**
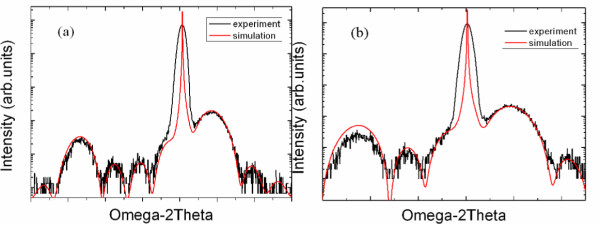
**The ω-2θ scans of SL structures**. (**a**) and (**b**) are the Images of SL structures with different Al compositions and periods. Red lines, experimental values; black lines, simulation values.

As shown in Figure 2, the simulation line is fitting well with the experiment line so we can exactly determine the thicknesses of AlN and GaN. The satellite peaks which are related with the diffraction of SLs can be seen in each picture indicating that the period of SLs is uniform and the interface is smooth.

Three groups of hetero-structures were grown. One group consisted of different Al compositions, and the other two consisted of equivalent Al compositions. While the SLs in the second group had different period thicknesses, the last group had the same period (3.5 nm) but with different numbers of SLs (4 to 6 periods). Hall effect measurements were performed using the vander Pauw method on cleaved 6-mm × 6-mm squares using In/Ga alloy as ohmic contacts.

## Results and discussion

**Figure 3:**
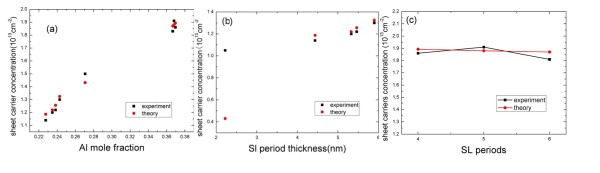
**Relations of sheet carrier concentrations**. Relations with (**a**) Al mole fraction, (**b**) SL period thickness, and (**c**) SL periods. Black filled squares, experimental values; red diamonds, theoretical values.

In the first group of hetero-structures, the nominal equivalent Al composition of the quasi-alloy varies from 22% to 40%. Figure 3a shows that sheet carrier density increased rapidly as the nominal equivalent Al composition increased. In the second group, the nominal Al compositions of the quasi-alloy are all 25%, and the sheet carrier density as a function of the SL period thickness is plotted in Figure 3b. The sheet carrier densities are increasing, with the SL period getting thick. In the third group, the AlN/GaN SL barrier had the same Al composition (Al% = 36.5%) and thickness of one SL period. As shown in Figure 3c, the sheet carrier densities are about the same when the periods of SLs increase. In other words, when the AlN/GaN SLs are under full strain, the sheet carrier densities are independent of the total thickness of AlN/GaN SLs. This is different from the situations in traditional AlGaN barrier HEMTs where the sheet carrier density is influenced by the Al composition and thickness of AlGaN. In the case of HEMT with the AlN/GaN SL barrier, we find that the sheet carrier densities are increasing with the period thickening of SLs, and yet, Al composition is still independent of the total thickness of AlN/GaN SLs.

In traditional AlGaN barrier HEMT structures, we can calculate the 2-DEG density using Equations 2 and 3 [9,10]:

(2) $$\left\{\begin{array}{c} n_{s}=\frac{\sigma }{e}\left(1-\frac{d_{0}}{d_{\text{AlGaN}}}\right)\\ d_{0}=\frac{\varepsilon_{0}\varepsilon_{\text{AlGaN}}}{\sigma e}\left(e\varphi_{B}+E_{\text{F}}-\Delta E_{\text{c}}\right) \end{array}\right)$$

where *ε*_0 _is vacuum permittivity, *n*_s _is the sheet carrier density of HEMT, *d*_0 _is the critical thickness of AlN which can form 2-DEG. If the thickness of AlN is smaller than *d*_0_, it could not form the 2-DEG. *ε*_AlGaN _is the relative dielectric constant of AlGaN, *d*_AlGaN _is the thickness of the barrier, e*φ*_B _is the Schottky barriers of the gate contact on top of AlGaN, *E*_F_(*x*) is the Fermi level with respect to the GaN conduction-band-edge energy, and Δ*E*_C _is the conduction band offset at the AlGaN/GaN interface where 2-DEG forms.

**Figure 4:**
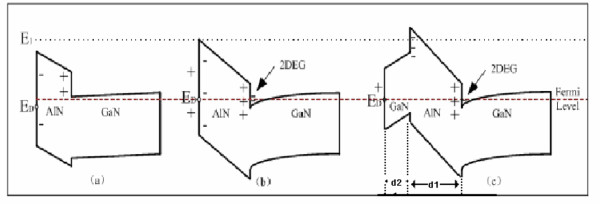
**The 2-DEG between AlN/GaN interface with different AlN thickness**. (a) The thickness of AlN barrier less than the critical thickness (*d*_0_), (b) the thickness of AlN barrier beyond *d*_0_, and (c) the thickness of AlN barrier beyond *d*_0_, and the GaN was deposited on the AlN barrier.

When AlN is deposited on GaN, it will produce negative (acceptor-like) piezoelectric polarization (PZ) charge density at the top of the AlN barrier layer and positive PZ charge density at the AlN/GaN interface [8] as shown in Figure 4 (a). There is no 2-DEG until the thickness of AlN barrier is beyond *d*_0 _which can be determined by Equation 2.

The energy *E*_D _of surface state is below the *E*_F _if the thickness of AlN is less than *d*_0_. With barrier thickness increasing, *E*_F_-*E*_D _is decreasing. In addition, the energy *E*_1_, the surface energy of conduction band, increases with increasing barrier thickness because there is a constant electric field in the AlN barrier due to the unscreened polarization field. At *d*_0 _(*E*_F_-*E*_D _= 0), the donors at a free surface will begin to be ionized, and the PZ charge density at the AlN/GaN interface will start to be compensated by the 2-DEG formed at the interface. The charge at the top of the AlN will be compensated by charged surface states for a free surface when the thickness of AlN barrier is beyond the critical thickness. At critical thickness (*E*_F_-*E*_D _= 0), electrons are able to transfer from occupied surface state to empty conduction band states at the interface. The 2-DEG density is increasing with increasing barrier thickness, while the *E*_D _and *E*_1 _are constant and independent on the barrier thickness. The *E*_1 _is dependent on the material of the barrier layer.

Base on these analyses, the interfacial charge between AlN and GaN that determines the density of 2-DEG is mainly dependent on the polarized intensity. For this reason, we calculate the 2-DEG density with AlN barrier according to Equation 2, and the result is shown in Equation 3 [9-11]:

(3) $$\left\{\begin{array}{c} \frac{\sigma }{e}=-P_{\text{SP}}^{\text{AlN}}-P_{\text{PZ}}^{\text{AlN}}+P_{\text{SP}}^{\text{GaN}}\\ n_{s}=\frac{\sigma }{\text{e}}\left(\text{1}-\frac{d_{\text{0}}}{d_{\text{AlN}}}\right)\\ d_{0}=\frac{\varepsilon_{\text{0}}\varepsilon_{\text{AlN}}}{\sigma \text{e}}\left(\text{e}\varphi_{\text{B}}+E_{\text{F}}-\Delta E_{\text{c}}\right) \end{array}\right)$$

where $P_{\text{SP}}^{\text{AlN}}$and $P_{\text{SP}}^{\text{GaN}}$are the spontaneous polarization of AlN and GaN, and $P_{\text{PZ}}^{\text{AlN}}$is the piezoelectric polarization of AlN buffer layer; $\frac{\sigma }{e}$ is the polarization-induced charge density determined by the difference in the total spontaneous and piezoelectric polarization within AlN and GaN layers; *n*_s _is the sheet carrier density; and *d*_0 _is the critical thickness of AlN which can form 2-DEG.

The 2-DEG could also influence *E*_F_; however, according to Equation 4 [12], we find that the change of *E*_F _is less than 10% even if the *n*_s _has a change of 10^12 ^when the sheet carrier density is up to 10^13 ^cm^-2^. In order to simplify the computing, we ignore the variation of *E*_F _coursed by 2-DEG:

(4) $$\left\{\begin{array}{c} E_{\text{F}}=E_{\text{0}}\left(x\right)+\frac{\pi \hbar^{\text{2}}}{m\text{*}\left(x\right)}\frac{n_{\text{s}}\left(x\right)}{\varepsilon \text{(}x\text{)}}\\ E_{\text{0}}\left(x\right)={\left\{\frac{\text{9}\pi \hbar {\text{e}}^{\text{2}}}{\text{8}\varepsilon_{\text{0}}\sqrt{\text{8}m*\left(x\right)}}\frac{n_{\text{s}}\left(x\right)}{\varepsilon \left(x\right)}\right\}}^{\text{2}/\text{3}} \end{array}\right)$$

However, when GaN is deposited on the AlN barrier, it can change the energy band as shown in Figure 4 (c). The surface state is pined on the energy *E*_F _because the thickness of AlN barrier is beyond *d*_0_. The energy *E*_1 _rises with the increasing thickness of GaN since the electric field also rises from the 2-DEG has not been screened. According to the equilibrium of total electric field, we take a simple electro-static analysis on the energy band and gain the result as shown in Equations 5 and 6:

(5) $$\left\{\begin{array}{c} \frac{\frac{\sigma }{\text{e}}-n_{\text{s}}}{\varepsilon_{\text{1}}}d_{\text{1}}=\frac{E_{\text{1}}-\Delta E_{\text{c}}+E_{\text{F}}}{\text{e}}\\ E_{\text{1}}=E_{\text{D}}+\frac{en_{\text{s}}}{\varepsilon_{\text{2}}}d_{\text{2}}+\Delta E_{\text{c}} \end{array}\right)$$

By speculating the equation, the density of 2-DEG can be expressed as:

(6) $$\begin{array}{c} n_{\text{s}}=\frac{\sigma }{\text{e}}\frac{\varepsilon_{\text{2}}d_{\text{1}}}{\varepsilon_{\text{2}}d_{\text{1}}+\varepsilon_{\text{1}}d_{\text{2}}}\left(\text{1}-\frac{\left(E_{\text{D}}+E_{\text{F}}\right)\varepsilon_{\text{1}}\varepsilon_{\text{2}}}{\sigma \text{e}\varepsilon \text{2}d\text{1}}\right)\\ \text{or}\\ n_{\text{s}}=\frac{\sigma }{\text{e}}\frac{\varepsilon_{\text{2}}d_{\text{1}}}{\varepsilon_{\text{2}}d_{\text{1}}+\varepsilon_{\text{1}}d_{\text{2}}}\left(\text{1}-\frac{\left(\text{e}\varphi_{\text{B}}+E_{\text{F}}\right)\varepsilon_{\text{1}}\varepsilon_{\text{2}}}{\sigma \text{e}\varepsilon_{\text{2}}d_{\text{1}}}\right) \end{array}$$

where *d*_1 _and *d*_2 _are the thicknesses of AlN barrier and GaN layer, respectively; *E*_F _is the Fermi level position with respect to the GaN conduction-band edge; and *ε*_1 _and *ε*_2 _are the di-electric constants of AlN and GaN, respectively. According to Equation 6, the 2-DEG density is dependent on both the Al mole fraction and the thickness of SL period but independent on the total thickness of SLs when the AlN/GaN SLs are used as the barrier layer.

In fact, the sheet carrier density measured using Hall coefficient is the average value of all SLs. It is difficult to calculate the 2-DEG density in every SL period directly. The sandwich structure shown in Figure 4 (c) can be seen as one period in SL barrier. We consider that the 2-DEG will be formed in the every SL period, and the sheet carrier density is equal in every SL period since the SLs have periodicity and tunnel impenetration. So, the *n*_s _in each channel could be calculated using Equation 6. Therefore, we think that we can simply calculate 2-DEG density of the outermost SL period simply to represent the 2-DEG density in every SL.

As is shown in Figure 3, the red diamonds correspond to simulate value by means of Equation 6 where the surface barrier value e *φ*_B _is fixed at 0.55 eV, which was obtained by fitting the first experimental data points in Figure 3a. Although there was no report about the e*φ*_B _of In/Ga alloy on GaN as seen in Figure 3a, b, c, we can see that the simulate values are accordingly well with the experimental value; hence, we think that Equation 6 can be used to compute the 2-DEG density.

However, as shown in Figure 3b, the simulate values highly contradict with the experimental values. If the thickness of SL is small, the energy band bending in each SL period should not be the same, and the sheet carrier density is not equal in every SL period. As the interface of hetero-structure is about 5-Å thick, the interfacial influence may become notable as the thickness of one SL period decreases which makes it more difficult to compute for the 2-DEG density.

## Conclusions

AlN/GaN SLs as the barrier of HEMT were grown on semi-insulated GaN, and the formation of 2-DEG was researched particularly. The 2-DEG concentration, characterized by Hall effect measurements, was found to be as a function of AlN thickness in SL period but independent on the total thickness of SLs. The calculation with our innovative method have proven these laws. During the process of calculation, we consider that the 2-DEG would form in every SL period when the AlN barrier thickness in SL period was beyond the critical thickness, and concentration of the 2-DEG was equivalent in each period. However, once the AlN barrier thickness in SL period is smaller than the critical thickness, the 2-DEG would not be formed in any SL period according to the theory. Besides, the interfacial influence may become notable, which makes it much more difficult to calculate the density. The model we set has proved to be fitted well with the experimental result and can be used to calculate the 2-DEG density in HEMTs with SL barriers.

## Abbreviations

2-DEG: two-dimensional electron gas; HEMT: high-electron mobility transistor; MOCVD: metal-organic chemical vapor deposition; PZ: piezoelectric polarization; SLs: super-lattices.

## Competing interests

The authors declare that they have no competing interests.

## Authors' contributions

PX and YC contributed the main ideas of super-lattice structure design and the calculation of 2-DEG density. YJ, ZM, XW, ZD, and YL carried out the MOCVD growth, the X-ray diffraction, and the Hall effect measurements. HJ, WW, and HC gave important advices to the paper. All authors read and approved the final manuscript.
